# Application of MALDI-TOF MS to Identify and Detect Antimicrobial-Resistant *Streptococcus uberis* Associated with Bovine Mastitis

**DOI:** 10.3390/microorganisms12071332

**Published:** 2024-06-29

**Authors:** Tingrui Zhang, Duangporn Pichpol, Sukolrat Boonyayatra

**Affiliations:** 1 Department of Food Animal Clinic, Faculty of Veterinary Medicine, Chiang Mai University, Chiang Mai 50100, Thailand; tingrui_z98@163.com; 2 Department of Veterinary Public Health, College of Veterinary Medicine, Yunnan Agricultural University, Kunming 100191, China; 3 Department of Veterinary Biosciences and Veterinary Public Health, Faculty of Veterinary Medicine, Chiang Mai University, Chiang Mai 50100, Thailand; d.pichpol@cmu.ac.th; 4 Department of Veterinary Clinical Sciences, College of Veterinary Medicine, Long Island University, Brookville, NY 11548, USA

**Keywords:** bovine mastitis, *Streptococcus uberis*, MALDI-TOF MS, antimicrobial resistance

## Abstract

*Streptococcus uberis* is a common bovine mastitis pathogen in dairy cattle. The rapid identification and characterization of antimicrobial resistance (AMR) in *S. uberis* plays an important role in its diagnosis, treatment, and prevention. In this study, matrix-assisted laser desorption ionization time-of-flight mass spectrometry (MALDI-TOF MS) was used to identify *S. uberis* and screen for potential AMR biomarkers. *Streptococcus uberis* strains (*n* = 220) associated with bovine mastitis in northern Thailand were identified using the conventional microbiological methods and compared with the results obtained from MALDI-TOF MS. *Streptococcus uberis* isolates were also examined for antimicrobial susceptibility using a microdilution method. Principal component analysis (PCA) and the Mann–Whitney U test were used to analyze the MALDI-TOF mass spectrum of *S. uberis* and determine the difference between antimicrobial-resistant and -susceptible strains. Using MALDI-TOF MS, 73.18% (161/220) of the sampled isolates were identified as *S. uberis*, which conformed to the identifications obtained using conventional microbiological methods and PCR. Using PCR, antimicrobial-resistant strains could not be distinguished from antimicrobial-susceptible strains for all three antimicrobial agents, i.e., tetracycline, ceftiofur, and erythromycin. The detection of spectral peaks at 7531.20 *m/z* and 6804.74 *m/z* was statistically different between tetracycline- and erythromycin-resistant and susceptible strains, respectively. This study demonstrates a proteomic approach for the diagnosis of bovine mastitis and potentially for the surveillance of AMR among bovine mastitis pathogens.

## 1. Introduction

Bovine mastitis is a major cause of economic losses in the dairy industry. The costs associated with bovine mastitis are estimated to be over USD 2 billion annually [1]. These costs come directly from milk discard, drugs used for treatment, veterinary services, and labor and indirectly from reduced milk production, premature culling, and the replacement of infected cows [2,3]. Bovine mastitis is also known to be associated with low milk quality, such as reducing casein, which induces the unpleasant smell in fresh milk and decreases curding and yield of cheese, consequently reducing the sale value of milk [4]. Moreover, milk and dairy products derived from udders with bovine mastitis, which is usually caused by intramammry infection (IMI) by a pathogen, have a higher risk of causing food poisoning, being contaminated with antibiotic residues, or carrying antimicrobial resistant microorganisms which can adversely affect consumers’ health [4].

Bovine mastitis is usually caused by intramammary infection by a pathogen. Once the pathogen has infected the mammary gland, it can induce the accumulation of leukocytes, releasing chemokines and cytokines, and consequently causing inflammation and tissue damage to the mammary gland. The severity of inflammation can vary from case to case and can involve udder swelling, pain and abnormal milk containing flakes, clots or blood [5]. Therefore, effective prevention and treatment programs to tackle bovine mastitis are very important for both animal well-being and the financial aspect of dairy farming. 

*Streptococcus uberis* is a common cause of bovine mastitis in Thailand [6] and other countries worldwide [7,8]. It has been described as an environmental pathogen. However, several studies have provided evidence supporting the ability of some strains of *S. uberis* to be transmitted from cow to cow [9,10]. To identify *S. uberis*, conventional microbiological culture and biochemical tests are generally used. However, these tests are time-consuming and cannot accurately differentiate *S. uberis* from closely related streptococci [11,12]. High-throughput technology that can efficiently identify *S. uberis* is useful for the development of the specific application of management control measures and treatment protocols [13].

Antimicrobial therapy is typically administered to treat bovine mastitis. Several antimicrobial agents have been reported for the treatment of intramammary infections caused by *S. uberis*. However, the success of the treatment of clinical bovine mastitis caused by *S. uberis* varies. A previous study reported the use of a 2-day treatment with pirlimycin to treat mastitis caused by *S. uberis,* but the clinical cure rate was only 50%, much lower than that of *Streptococcus dysgalactiae* (100%), using a similar treatment protocol [14]. Other studies have reported a cure rate of only 44% when treating *S. uberis* IMI with penicillin [15] or 50–58% for a 5-day treatment with benzylpenicillin, which are unsatisfactory treatment responses [16]. 

The reduced success rate of antibiotic treatment for *S. uberis* IMI may be associated with the antimicrobial resistance (AMR) of this pathogen. Previous studies have suggested high resistance to some commonly used antibiotics among bovine mastitis pathogens, including *S. uberis* [17,18]. Moreover, several studies have reported the presence of various AMR genes, such as the lincomycin resistance gene (*lnu*D), erythromycin resistance gene (*erm*B), and macrolide resistance gene (*mph*(B)) in *S. uberis* [19,20,21]. This evidence emphasizes the AMR development issue in *S. uberis*.

Matrix-assisted laser desorption ionization–time of flight mass spectrometry (MALDI-TOF MS) is a proteomic approach to rapidly identify disease pathogens, particularly bacteria. This technique has been used in various fields, including bacterial detection, medicine, food safety, and public health [22]. Compared to the conventional bacterial identification, using MALDI-TOF MS can give results at least 1 day earlier, has lower marginal costs, and requires simpler procedures and fewer reagents [23]. A previous study used MALDI-TOF MS to identify *Streptococcus agalactiae* isolates based on different phylogenetics [24]. Another study demonstrated high accuracy in the identification of group D streptococci in bovine mastitis cases [25]. In 2018, Esener et al. used MALDI-TOF MS and machine learning to discriminate between contagious and environmental strains of *S. uberis* isolated from dairy farms [26]. Recently, MALDI-TOF MS was successfully used to detect mastitis pathogens in bovine milk samples [27] and antimicrobial resistance in *Staphylococcus aureus* associated with bovine mastitis [28]. These studies emphasize the potential of MALDI-TOF MS applications, which can dramatically improve the accuracy and speed of the diagnosis, epidemiological investigation, and monitoring of AMR of bovine mastitis pathogens.

In this study, we aimed to apply MALDI-TOF MS technology to characterize *S. uberis* associated with bovine mastitis in northern Thailand, and to identify certain mass peak patterns or biomarkers associated with AMR as determined by a microdilution method. 

## 2. Materials and Methods

### 2.1. Streptococcus uberis Isolates

*Streptococcus uberis*, previously isolated from milk samples submitted to the central laboratory of the Faculty of Veterinary Medicine, Chiang Mai University, for the diagnosis of clinical and subclinical bovine mastitis from January 2012 to December 2017, were included in the study. All *S. uberis* isolates were frozen at −80 °C in brain heart infusion broth (Merck^®^, Darmastadt, Germany) containing 20% glycerol until use. In total, 220 frozen *S. uberis* isolates were randomly selected for use in this study. The frozen isolates were thawed at room temperature, re-grown on 5% bovine blood agar (Merck^®^, Darmastadt, Germany), and incubated at 37 °C for 24 h to 48 h. Biochemical tests, including Gram staining, catalase tests, and metabolization test of esculin, inulin, mannitol, and salicin, were used to presumptively identify *S. uberis*, as suggested by the National Mastitis Council [29]. 

Genomic DNA was extracted from all the isolates using a DNA extraction kit (NucleoSpin^®^, Düren, Germany). The PCR amplification of the species-specific *16s r*RNA gene was used to confirm the identity of all *S. uberis* isolates, as previously described [10]. Briefly, the PCR mixture (25 µL) containing 0.5 µL of forward (5′-CGCATGACAATAGGGTACA-3′) and reverse (5′-GCCTTTAACTTCAGACTTATCA-3′) primers (10 mol/L); 12.5 µL of 2× Taq Master Mix contained 1.25 U of Taq DNA polymerase, 1× ViBuffer A, 0.2 mM dNTPs, and 1.5 mM MgCl_2_ (MyTaqTM Red Mix, Bioline, Australia); 11 µL of DNase-free water; and 0.5 µL of DNA template (50–100 ng/µL) was prepared for each PCR reaction of each isolate. The PCR reaction was performed in a thermal cycler with the following program: initial denaturation at 94 °C for 60 s, 30 cycles of denaturation at 94 °C for 60 s, annealing at 58 °C for 90 s, and extension at 72 °C for 90 s. The PCR products were separated by gel electrophoresis in 2% agarose gel and stained with ethidium bromide. To visualize the PCR products, the stained agarose gels were examined under an ultraviolet transilluminator system (Bio-Rad Laboratories, Hercules, CA, USA). The PCR product with the size of 445 bp was expected for the identification of *S. uberis*. The PCR products were further sequenced to confirm their identifications.

### 2.2. Determination of the Antimicrobial Susceptibility of S. uberis Isolates

The antimicrobial susceptibility of all *S. uberis* isolates was determined by examining the minimum inhibitory concentration (MIC) of antimicrobial agents required to inhibit bacterial growth using the microdilution method, as recommended by the Clinical and Laboratory Standards Institute (CLSI) performance standards [30]. Five antimicrobial agents, penicillin, ceftiofur, erythromycin, tetracycline, and gentamycin, were used for MIC determination. The antimicrobial agents were selected as the representatives of five common classes of antimicrobial agents used in dairy cattle farms in the region [31]. These classes included penicillins, cephalosporins, macrolides, tetracyclines, and aminoglycosides. To perform the broth microdilution assay, a stock solution of each antimicrobial agent was diluted in double Mueller–Hinton broth (MHB) (Merck^®^, Darmastadt, Germany) to prepare the working solution. A two-fold dilution covering the diluted concentration range of each antimicrobial agent was performed for each working solution on a 96-well microtiter plate. The selected diluted concentration ranges were 0.0625–256 µg/mL for ceftiofur, 0.0039–4 µg/mL for penicillin G, 0.625–64 µg/mL for tetracycline, 0.0039–8 µg/mL for erythromycin, and 0.0039–16 µg/mL for gentamycin. Fresh colonies of each *S. uberis* isolate were cultured in the MHB medium. The inoculated MHB turbidity was adjusted to the 0.5 McFarland standard (approximately 1 × 10^8^ colony-forming units/mL) using a McFarland densitometer (DEN-1^®^, Biosan, Riga, Latvia). A total of 90 microliters of the suspension was transferred to 9 mL of normal saline solution and mixed thoroughly. Fifty microliters of the bacterial suspension was dispensed into each well of the diluted antimicrobial agents. The plates were then incubated at 37 °C for 20 h. *Streptococcus pneumoniae* ATCC^®^ 49619 and *Esherichia coli* ATCC^®^ 25922 were used as quality control strains. The MICs were determined as the lowest antimicrobial concentrations that inhibited visible bacterial growth. The antimicrobial susceptibility of each isolate was determined as ‘resistance’ (R), ‘intermediate’ (I), or ‘susceptible’ (S) using the breakpoint standards listed in Table 1.

### 2.3. MALDI-TOF MS Analysis for S. uberis

All selected strains were analyzed by MALDI-TOF MS (Micoroflex LT system, Bruker Daltonics, Bremen, Germany). An overnight culture of *S. uberis* grown on blood agar (Merck^®^, Darmastadt, Germany) was used. The direct colony-on-plate extraction method was adopted, as previously described by Alatoom et al. [33]. A single colony of each isolate was randomly selected and applied to a 96-spot stainless steel target plate. Subsequent to drying, the inoculated spots were overlaid with 1 µL of the matrix solution, which was composed of a saturated solution of α-cyano-4 hydroxycinnamic acid 10 mg/mL in 50% acetonitrile (Sigma-Aldrich^®^, Merck Ltd., Darmstadt, Germany) and 2.5% trifluoroacetic acid (Sigma-Aldrich^®^, Merck Ltd., Darmstadt, Germany). The spots were allowed to dry completely, and the target plate was inserted into the MALDI-TOF MS system. 

MALDI-TOF MS data were retrieved by analyzing samples using MALDI Biotype 3.0 software for spectral profile matching with the MALDI Biotyper^®^ Library database (Bruker Daltonics, Bremen, Germany). The output was reported as a log score with a maximum value of 3.0. Scores between 2.0 and 3.0 indicated that the species-level identification was reliable. Scores between 1.7 and 1.9 indicated that the genus-level identification was reliable, but the species-level identification was questionable. Scores lower than 1.7 indicated unreliable identification. 

### 2.4. MALDI-TOF MS Spectra and Statistical Analyses

The spectrum data were processed and analyzed using BioNumerics 8.1 (Applied Maths NV, Sint-Martens-Latem, Belgium). The raw spectra were imported into a database and preprocessed by trimming the x-axis to a minimum of 2000 *m/z* without an upper limit. The entire preprocessing workflow was then executed for peak detection using the default signal-to-noise ratio. After preprocessing the spectra data, peak matching was performed with a constant tolerance of “1.9”, a linear tolerance of 550, and a peak detection rate of 10%. Principal component analysis (PCA) of the peak classes was applied to reduce the complexity of the data and identify groups by visualizing the data in two or three dimensions. 

Peak class data of “R” and “S” isolates of each antimicrobial agent were statistically compared as independent tests using Mann–Whitney U test. All peak classes with a *p*-value lower than 0.05 (*p* < 0.05) were considered to be significantly different between “R” and “S” isolates.

## 3. Results

**Figure 1:**
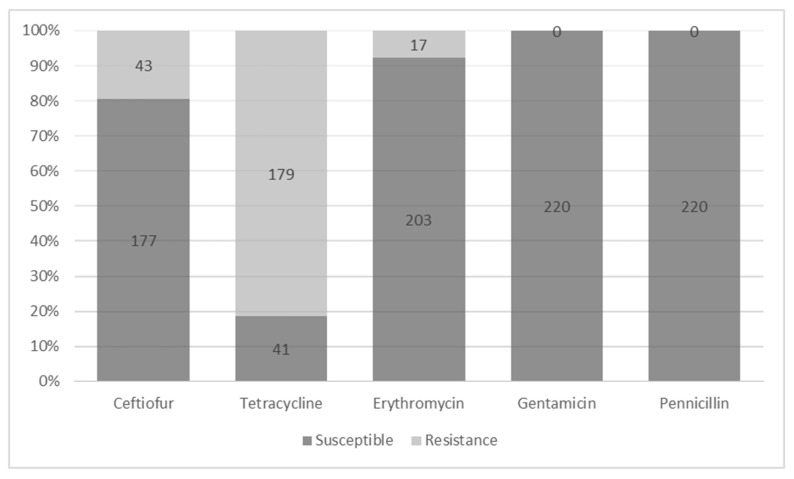
Antimicrobial susceptibility of *Streptococcus uberis* isolates associated with bovine mastitis in northern Thailand (*n* = 220).

A total of 220 isolates were retrieved and confirmed as *S. uberis* using conventional biochemical tests and PCR. Most *S. uberis* isolates were shown to be resistant to tetracycline (178/220, 80.91%), followed by ceftiofur (42/220, 19.09%) and erythromycin (17/220, 7.73%), as shown in Figure 1. All the examined *S. uberis* isolates were susceptible to penicillin G and gentamycin. 

All 220 *S. uberis* isolates were analyzed using MALDI-TOF MS. According to the score indicated in the MALDI Biotyper output, 23 isolates showed scores of <0 without any spectrum peak detected, and 28 isolates showed scores between 0 and 1.7, indicating an unreliable identification. In addition, eight isolates were identified as species other than *S. uberis,* including *Enterococcus faecium* (*n* = 1), *Enterococcus faecalis* (*n* = 2), *Enterococcus pseudoavium* (*n* = 1), *Streptococcus lutetiensis* (*n* = 1), *S. dysgalactiae* (*n* = 1), and *Lactococcus garvieae* (*n* = 2). The isolates with low scores (*n* = 51) and isolates identified as other species (*n* = 8) were excluded from MALDI-TOF MS spectral analyses. Among the remaining 161 isolates, 112 and 49 were identified as *S. uberis* with log scores of ≥2 and between 1.7 and 2.0, respectively. These 161 *S. uberis* isolates were included in the MALDI-TOF MS spectral analysis.

**Figure 2:**
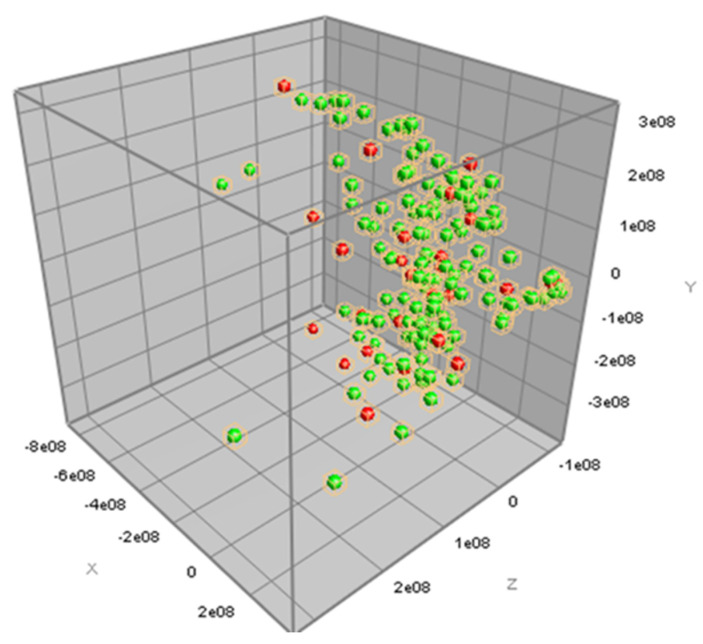
Three-dimensional view of the principal component analysis (PCA) of MALDI-TOF MS data for ceftiofur susceptibility. Spectral data of ceftiofur-susceptible (green dot) and ceftiofur-resistant (red dot) *Streptococcus uberis* isolates (*n* = 161) are plotted with three most descriptive principal components.

**Figure 3:**
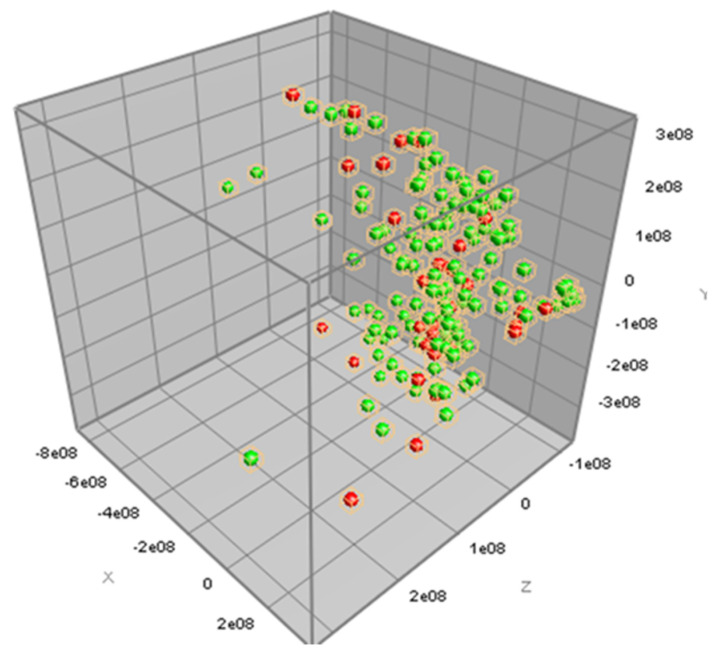
Three-dimensional view of the principal component analysis (PCA) of MALDI-TOF MS data for tetracycline susceptibility. Spectral data of tetracycline-susceptible (red dot) and tetracycline-resistant (green dot) *Streptococcus uberis* isolates (*n* = 161) are plotted with three most descriptive principal components.

**Figure 4:**
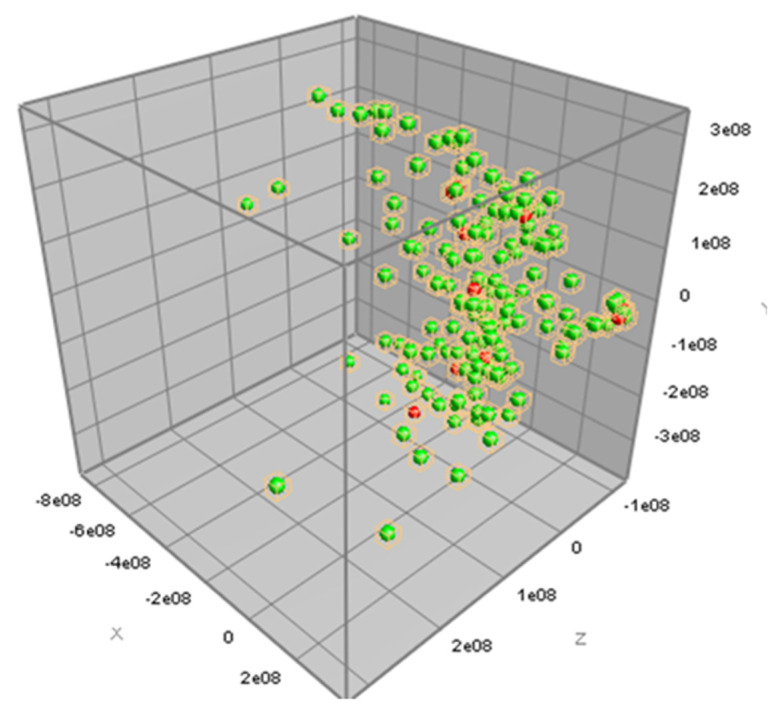
Three-dimensional view of the principal component analysis (PCA) of MALDI-TOF MS data for erythromycin susceptibility. Spectral data of erythromycin-susceptible (green dot) and erythromycin-resistant (red dot) *Streptococcus uberis* isolates (*n* = 161) are plotted with three most descriptive principal components.

The information of antimicrobial susceptibility according to the MIC results of the three antimicrobial agents, tetracycline, ceftiofur, and erythromycin, together with the peak class data from the MALDI-TOF MS spectra results of 161 *S. uberis* isolates, were analyzed for PCA. The antimicrobial-resistant strains could not be distinguished from the antimicrobial-susceptible strains for all three antimicrobial agents using PCA, as illustrated in Figure 2, Figure 3 and Figure 4. 

**Figure 5:**
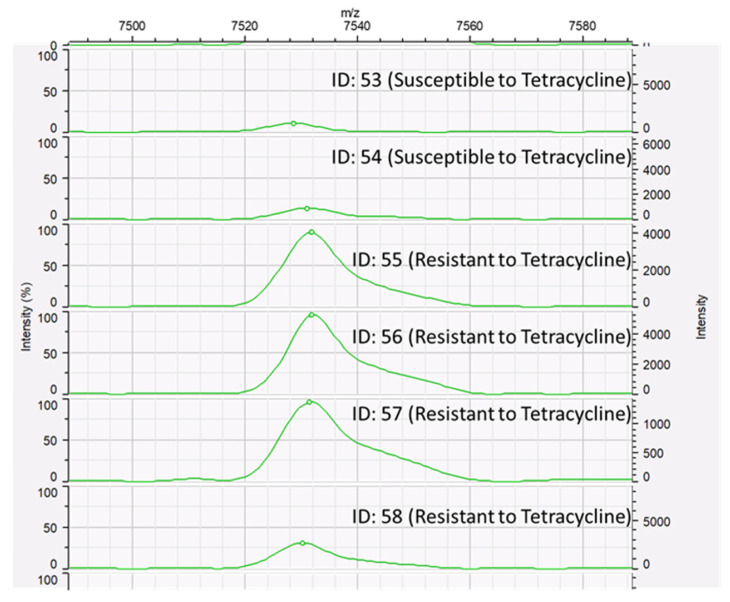
MALDI-TOF spectra of *Streptococcus uberis* isolates with tetracycline susceptibility. Tetracycline-susceptible strains (ID: 53 and 54) and tetracycline-resistant strains (ID: 55, 56, 57, and 58) to tetracycline. A peak of 7531.20 *m/z* was detected among tetracycline-resistant *S. uberis* isolates.

**Figure 6:**
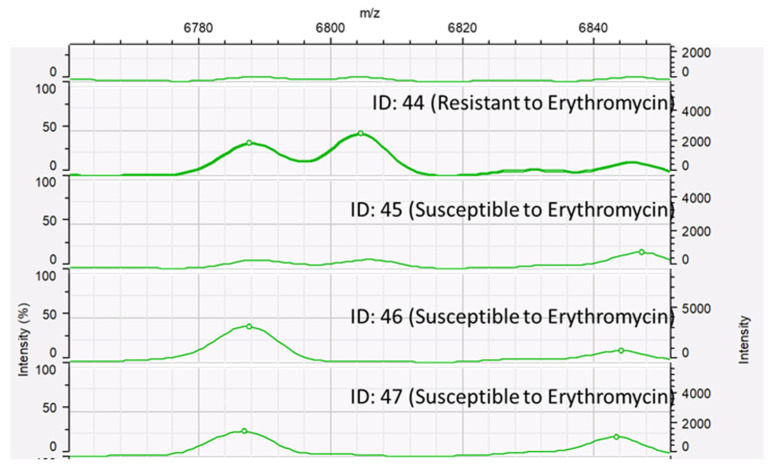
MALDI-TOF spectra of *Streptococcus uberis* isolates with erythromycin susceptibility; isolates were susceptible (ID: 45, 46, and 47) and resistant (ID: 44) to erythromycin. A peak of 6804.74 *m/z* was detected with erythromycin-resistant *S. uberis* isolates.

The peak class data of resistant and susceptible isolates for each antimicrobial agent were statistically compared using the Mann–Whitney U test. No statistically significant peaks were detected among ceftiofur-resistant and ceftiofur-susceptible *S. uberis* isolates. The detection of the spectral peak at 7531.20 *m/z* was statistically different between tetracycline-resistant and tetracycline-susceptible *S. uberis* isolates (*p* = 0.046; *p* < 0.05), as shown in Figure 5. The detection of the spectral peak at 6804.74 *m/z* was statistically different between the erythromycin-resistant and erythromycin-susceptible *S. uberis* isolates (*p* = 0.019; *p* < 0.05), as shown in Figure 6.

## 4. Discussion

*Streptococcus uberis* is one of the most common pathogens associated with bovine mastitis [14]. Several antimicrobial agents, such as cephalosporins, β-lactams, macrolides, and lincosamides have been applied to treat *S. uberis* IMI [34]. However, the cure rate of antimicrobial treatment of bovine mastitis caused by *S. uberis* infection varies from 44 to 79% [15,35]. This variation can be associated with the AMR of this pathogen.

In this study, we found that most *S. uberis* isolates from northern Thailand were resistant to tetracycline, less than 20% were resistant to ceftiofur and erythromycin, and none were resistant to gentamicin and penicillin G. The high prevalence of tetracycline resistance in *S. uberis* isolates from bovine mastitis cases is common worldwide [18,36,37]. However, high variation in resistance to other antimicrobial agents has been reported. One study revealed a low prevalence of erythromycin resistance (9%) among *S. uberis* in central California [36], whereas another study demonstrated a much higher prevalence of erythromycin resistance (60%) among *S. uberis* isolates from multiple regions in the USA [38]. A recent study in Brazil reported a high resistance rate to erythromycin (80.7%) and penicillin G (57.8%) and a low resistance rate to ceftiofur (10.8%) among *S. uberis* isolates [18]. The variation in antimicrobial resistance of *S. uberis* can be influenced by differences in antimicrobial use on dairy farms in different countries. The long-term treatment of *S. uberis* with antibiotics may induce resistance, which can increase the difficulty of treatment and is associated with public health concerns [39,40]. 

MALDI-TOF MS is widely used for veterinary diagnosis and microbiological identification. Several studies have introduced the use of MALDI-TOF MS for the identification and AMR determination of pathogens that cause bovine mastitis [28]. Several studies have reported high accuracies in identifying both Gram-positive and Gram-negative bacteria associated with bovine mastitis using MALDI-TOF MS, ranging from 93.5% to 100% [41,42]. A recent study by Jahan et al. reported a lower agreement between MALDI-TOF MS and *16S r*DNA sequencing results for the diagnosis of bovine mastitis pathogens at the species level to be 74% and at the genus level to be 93% [27]. However, these previous reports did not specifically study the identification of *S. uberis* isolates. Our results showed that only 73.18% (161/220) of the sampled *S. uberis* isolates, as confirmed by conventional microbiological methods and PCR, were identified as *S. uberis* by MALDI-TOF MS. Considering the reliability of the results based on the interpretation of log scores from the MALDI-TOF MS output, only 50.91% (112/220) of *S. uberis* isolates were correctly identified at the species level, while 161 isolates (73.18%) were correctly identified at the genus level. The lower accuracy of the MALDI-TOF MS technique in this study compared to other previous studies could be due to the different method used for sample preparation and the limitations of the MALDI-TOF Biotyper database used for *S. uberis* identification.

The misidentification of bacterial pathogens using MALDI-TOF MS could be affected by the different methods used for sample preparation. Two methods are used for MALDI-TOF MS bacterial identification: the intact-cell method (ICM) or the direct colony method, and the protein extraction method (PEM) [43]. In this study, ICM was used for preprocessing *S. uberis* isolates because it is simple, fast, and routinely used for sample preparation in our laboratory. Although both ICM and PEM have been regularly used for Gram-positive and Gram-negative bacteria, ICM has been reported to be less accurate than PEM for identifying Gram-positive cocci [33]. The accuracy of identifying Gram-positive cocci when ICM was used to prepare samples for MALDI-TOF MS was as low as 63% at the genus level and 26% at the species level, compared with 98% and 79% at the genus and species levels, respectively, when PEM was used [30]. Therefore, the interpretation of MALDI-TOF MS data for *S. uberis* when ICM is used for sample preparation should be performed cautiously. 

In addition to sample preparation techniques, the limitations of the MALDI-TOF Biotyper database can influence the accuracy of bacterial identification. Although there are more than 7000 reference spectra in the MALDI-TOF Biotyper database, the spectra of the same species can differ depending on the source of the isolates [44]. Information on the spectra of bacteria isolated from milk samples can be minor compared to other sources, such as humans, the environment, and food. The bacterial isolates associated with bovine mastitis displayed different spectral patterns from the reference spectra stored in the database, and consequently had low log scores, resulting in unreliable or incorrect identification. Therefore, a customized spectral database of bovine mastitis-associated pathogens should be established and used to improve the accuracy of identification using MALDI-TOF MS. 

In this study, we analyzed the MALDI-TOF spectra to determine the AMR characteristics of *S. uberis*. PCA was used to analyze all the mass spectra and determine the differences in the spectra of the phenotypic resistance of *S. uberis* to the three antimicrobial agents. However, our results showed that the AMR characteristics were not distinguishable by PCA. In general, PCA is a preferred statistical technique for the analysis of mass spectrum. PCA can be used to extensively analyze the spectral data and create a profile consisting of some mass peaks potentially considered as biomarkers [45]. Because of the fact that our PCA analysis could not demonstrate any significant profile, our results might have limited clinical use and application. However, using another statistical comparison, we could identify one mass peak (7531.20 *m/z*) differently detected between tetracycline-resistant and tetracycline-susceptible *S. uberis* and another mass peak (6804.74 *m/z*) differently detected between erythromycin-resistant and erythromycin-susceptible *S. uberis*. These mass peaks explain the molecular weight of peptides differently expressed among the antimicrobial susceptible and resistant strains of *S. uberis*. Since these findings were a result of the Mann–Whitney U test, which did not analyze multiple mass peaks together as profiles, the interpretation for the use of these findings is cautious. 

Specific proteins and peptides associated with AMR phenotypes have been successfully detected in various microorganisms. Examples of successful experiments include the detection of carbapenemase production in *Bacteroides fragilis* using MALDI-TOF MS [46], and the detection of a phenol-soluble protein toxin (PSM-mec) associated with methicillin-resistant *S. aureus* (MRSA) [47]. More specific biomarker detection methods to characterize antimicrobial resistance in *S. uberis* should also be further investigated.

The peaks obtained from MALDI-TOF MS spectra can be useful in identifying the genus and species of microorganisms due to the unique protein mass fingerprints that different microorganisms exhibit. MALDI-TOF MS is highly effective for microbial identification at the genus and species level, because the characteristic mass spectra of ribosomal proteins and other abundant ‘structural’ proteins serve as unique fingerprints that can be matched against reference databases to accurately identify microorganisms [48]. However, estimating resistance to antibiotics by the direct detection of the AMR determinant based on these exhibited mass peaks is more complex and less direct. The difficulties of this application are due to the fact that proteins conferring AMR usually have large molecular weight which cannot be detected using the routine spectral range used for microbial identification [49]. Moreover, these produced AMR determinant proteins are usually less abundant in the cell compared to other ‘structural’ proteins and consequently have low peak intensities and are difficult to analyze [49]. While certain resistance mechanisms can lead to specific protein expression profiles or modifications that might be detected by MALDI-TOF MS, this method is generally not the standard for antibiotic resistance testing. Other techniques, such as PCR for resistance genes or phenotypic assays (e.g., disk diffusion, e-test, or broth microdilution), are typically used for this purpose. In summary, while MALDI-TOF MS is excellent for genus and species identification, additional methods are usually needed to reliably determine antibiotic resistance of microorganisms.

## 5. Conclusions

*Streptococcus uberis* isolates associated with bovine mastitis in northern Thailand were highly resistant to tetracycline, followed by ceftiofur and erythromycin. MALDI-TOF MS can be used to identify *S. uberis* isolates originated from milk samples of bovine mastitis cases. Regarding the application to differentiate the AMR characteristics among *S. uberis*, even though we could not detect some specific mass spectral profiles to be effectively used as biomarkers for this purpose, we could identify two mass peaks, one for tetracycline and the other for erythromycin, which were differently detected between the resistant and susceptible *S. uberis* strains. However, the use of a single mass peak for differentiation is not suggested. Although MALDI-TOF MS has been successfully used to identify bacteria in microbiological research, the detection of antibiotic resistance is still in its early stages of development. MALDI-TOF MS is a technology that is fast, simple to operate, and highly accurate. This technology can optimize the laboratory testing process, save detection time and cost, and aid in veterinary clinical diagnosis and treatment. It is essential to conduct further studies to characterize potential proteins or biomarkers through peptide mass fingerprinting and compare with established databases.

## Figures and Tables

**Table 1:** Concentration breakpoints to determine the antimicrobial susceptibility (S), intermediate (I), and resistance (R) of *Streptococcus uberis* recommended by the Clinical and Laboratory Standards Institute (CLSI).

Antimicrobial Agents	Breakpoint (μg/mL)			References
	S	I	R	
Penicillin G	≤0.12	0.25–2	≥4	[30]
Gentamycin	≤4	8	≥16	[32]
Erythromycin	≤0.25	0.5	≥1	[30]
Tetracycline	≤2	4	≥8	[30]
Ceftiofur	≤2	4	≥8	[30]

## Data Availability

The data presented in this work are available from the corresponding authors upon request.
